# Fluorometric In Situ Monitoring of an *Escherichia coli* Cell Factory with Cytosolic Expression of Human Glycosyltransferase GalNAcT2: Prospects and Limitations

**DOI:** 10.3390/bioengineering3040032

**Published:** 2016-11-21

**Authors:** Karen Schwab, Jennifer Lauber, Friedemann Hesse

**Affiliations:** Biberach University of Applied Sciences, Institute of Applied Biotechnology (IAB), 88400 Biberach, Germany; Lauber.Jennifer@web.de (J.L.); hesse@hochschule-bc.de (F.H.)

**Keywords:** *E. coli* SHuffle^®^ T7, Glycosyltransferase GalNAcT2, In Situ monitoring, soft sensor, fluorescence spectroscopy

## Abstract

The glycosyltransferase HisDapGalNAcT2 is the key protein of the *Escherichia coli* (*E. coli*) SHuffle^®^ T7 cell factory which was genetically engineered to allow glycosylation of a protein substrate in vivo. The specific activity of the glycosyltransferase requires time-intensive analytics, but is a critical process parameter. Therefore, it has to be monitored closely. This study evaluates fluorometric in situ monitoring as option to access this critical process parameter during complex *E. coli* fermentations. Partial least square regression (PLS) models were built based on the fluorometric data recorded during the EnPresso^®^ B fermentations. Capable models for the prediction of glucose and acetate concentrations were built for these fermentations with rout mean squared errors for prediction (RMSEP) of 0.19 g·L^−1^ and 0.08 g·L^−1^, as well as for the prediction of the optical density (RMSEP 0.24). In situ monitoring of soluble enzyme to cell dry weight ratios (RMSEP 5.5 × 10^−4^ µg *w/w*) and specific activity of the glycosyltransferase (RMSEP 33.5 pmol·min^−1^·µg^−1^) proved to be challenging, since HisDapGalNAcT2 had to be extracted from the cells and purified. However, fluorescence spectroscopy, in combination with PLS modeling, proved to be feasible for in situ monitoring of complex expression systems.

## 1. Introduction

The successful expression of target proteins that require post-translational modifications, such as glycosylations or disulfide bond formation, remains a challenge in *Escherichia coli*. These modifications are indispensable for protein folding, stability, and activity. Disulfide bond formation is usually compartmentalized in the periplasm of *E. coli*. The oxidizing environment and the presence of various chaperones in the periplasm enable the oxidation of sulfhydryl groups between two cysteine side chains resulting in a covalent disulfide bond [1,2]. Nevertheless, *E. coli* periplasm is poorly adapted for the production of multi-disulfide-bonded proteins in high yields since the periplasmic space is significantly smaller than the cytoplasmic space [3]. Therefore, another option is the expression of recombinant protein in the cytoplasm, whereby the chance for inclusion body formation is given. High expression rates and the lack of chaperones mediating correct folding and disulfide bond formation supports the accumulation of insoluble protein. Hence, some strains were engineered to enable the formation of disulfide bonds in the cytoplasm. These strains carry mutations in both the thioredoxin reductase (*trxB*) and the glutathione reductase (*gor*) genes to provide a less reducing cytoplasmic environment [1,4,5]. In addition to the mutations in *trxB* and *gor*, SHuffle^®^ T7 carries the chromosomally-integrated gene for the disulfide bond isomerase DsbC without the signal sequence [1]. To engineer an *E. coli* cell factory capable of glycosylating a protein substrate within the cytoplasm requires the expression of an additional enzyme. In the work presented here, the recently established *E. coli* cell factory derived from the SHuffle^®^ T7 strain was used. The proposed strain by Lauber et al. [6] was genetically engineered to express a functional recombinant human-derived glycosyltransferase. It could be shown that the co-expression of two redox folding helpers enabled the formation of a soluble enzyme with four disulfide bonds. This contributed to the development of a glycosylation system in *E. coli* for the transfer of a GalNAc-residue to a protein substrate in the cytoplasm [6]. The selection of the media used for bacteria fermentations is not negligible and can have a major impact on the bacterial cell factory [7]. High initial glucose concentrations in batch cultivations rapidly deplete accompanied by acetate increases at the same time [7]. Jain et al. showed in their experiments, that the glucose concentrations regulated the growth rates in *E. coli* fed-batch cultivations. Hoffmann et al. [8] decreased inclusion body formation in *E. coli* fed-batch cultures expressing β-galactosidase-HIVgp41 fusion protein by maintaining low glucose concentrations. Whereas Luchner et al. [9] controlled the expression rate of soluble human superoxide dismutase in *E. coli* by limiting the induction. The group showed that slowing down the protein expression shifts the ratio of soluble protein to inclusion bodies towards the soluble product. Moreover, Hortsch and Weuster-Botz [7] showed that the enzymatic glucose release of the EnPresso^®^ B medium can help to increase the expression of soluble formate dehydrogenase. They concluded that the consistently low glucose concentrations prevented the *E. coli* cells from metabolic overflow. Hence, the growth rates were reduced and the culture showed no acetate shoot up in combination with a pH drop.

Various process analyzers were already used over the last 15 years to set up non-invasive online monitoring systems for process parameters. Chemometric modeling was used to access the complex data structure generated with the used process analyzers [10,11,12]. Among the used methods, 2D fluorescence spectroscopy (2DFS) has been proven to be a highly valuable, sensitive, and reliable process analyzer which can be used for the prediction of substrate, product, and metabolite concentrations [9,13,14,15]. In general, principal components analysis (PCA) can be used to analyze the structure of the generated datasets, investigate multi-factorial relationships, and extract the relevant information [16,17]. Furthermore, partial least square regression (PLSR) can be applied to correlate offline measured process parameters, such as optical density or glucose concentrations (*y*-data), and the online recorded fluorescence scans (*x*-data) via linear regression [18]. Fluorescent components that are involved in cell growth and metabolism [19] change characteristically in the course of cultivation. The overall fluorescence signal of the bacterial culture is a mixture of fluorescence signals, which originate from components such as aromatic amino acids, ATP, NADH, FAD, and vitamins (riboflavin and pyridoxine). Furthermore, some fluorophores are also supplemented to the culture with the feed medium [14]. Cell growth and biomass formation are the most common monitored bioprocess parameters based on 2DFS. This was not only applied for *E. coli* cultivation processes [20], but also for *Saccharomyces cerevisiae* [21], *Pichia pastoris* [22], *Aspergillus niger* spores [23] and *Klebsiella pneumoniae* [24]. In addition, existing literature also shows that substrate, metabolite, and product concentrations [25,26] can be monitored in microbial cultivations but also in mammalian cultures [14,15]. Luchner et al. were even able to predict the concentration of soluble recombinant superoxide dismutase in *E. coli* fed-batch cultivations based on 2DFS [9]. The general attempt of all of these studies was to avoid complex and time consuming offline analyses, improve process understanding, and enable better process control.

The *E. coli* cell factory expressing soluble human-derived glycosyltransferase was chosen as the model system for this study. The cell factory expresses two helper proteins in order to support the formation of active HisDapGalNAcT2. Hence, the amount of functionally active enzyme in the cytoplasm was an indication of the cell factories performance. These process parameters were not accessible during the fermentation and had to be determined afterwards. The aim of this study was to evaluate if 2DFS based soft sensors can be applied for in situ prediction of difficult-to-access process parameters that require time-intensive and costly analytics. Furthermore, standard process parameters were monitored since a highly fluorescent medium with enzymatic glucose release was used, which was assumed to complicate the model generation. These models were built to illustrate the differences and challenges we were facing while calibrating PLSR models for the prediction of soluble protein to dry weight ratios and the specific activity of the glycosyltransferase. The study illustrated that it can be beneficial to use process analyzers to monitor all critical process parameters in real-time.

## 2. Material and Methods

### 2.1. Strain

All experiments were performed with *E. coli* SHuffle^®^ T7 (C3026H, New England Biolabs, Frankfurt am Main, Germany) expressing the recombinant human glycosyltransferase fusion protein HisDapGalNAcT2 and the chaperones Erv1p and PDI [6]. The glycosyltransferase HisDapGalNAcT2, which was encoded on the plasmid pET23d(+)::*HisDapGalNAcT2,* was under the control of the T7-promotor and construction of the plasmid was described previously [6]. The two chaperone genes on the plasmid pMJS9 were under the control of the arabinose promotor. Plasmid pMJS9 [27] was kindly provided by L. W. Ruddock. Unless otherwise stated, chemicals and reagents were obtained from Sigma-Aldrich (Taufkirchen, Germany) or Roth (Carl Roth GmbH and Co. KG, Karlsruhe, Germany).

### 2.2. Pre-Cultivation

The *E. coli* pre-cultures to inoculate the EnPresso^®^ B (BioSilta Oy, Oulu, Finland) batch process were cultivated in LB medium (120 μg·mL^−1^ ampicillin, 34 μg·mL^−1^ chloramphenicol and 0.2% glucose). The pre-cultures were inoculated with eight ceramic cryo beads containing the *E. coli* SHuffle^®^ T7 strain. The pre-cultures were grown in 50 mL Falcon^TM^ tubes (Fisher Scientific, Schwerte, Germany) containing 15 mL medium for 8 h at 37 °C/175 rpm.

### 2.3. Bioreactor Culture

A 2 L benchtop bioreactor BIOSTAT^®^ Bplus (Sartorius, Göttingen, Germany) equipped with two rushton impellers was used for the cultivation with a working volume of 1.4 L. Temperature, pH, and aeration were set to 30 °C, pH 7, and 0.05 vvm, respectively. The oxygen saturation was kept constant at 60% via agitation starting with a lower limit of 100 rpm. The pH was controlled with 1M NaOH and 1M H_2_SO_4_. Furthermore 5% (*v/v*) DOW CORNING^®^ medical antifoam (Dow Corning, Midland, MI, USA) was used.

### 2.4. Batch Cultivation with EnPresso® B Medium

The complex predefined EnPresso^®^ B medium including booster tablets was used as growth medium. The EnPresso^®^ B tablets were dissolved in sterile demineralized water and the solubilized medium was transferred into the sterilized bioreactor. Following the protocol provided by the manufacturer, the pre-culture was used for inoculation and a final OD_600_ of ≤0.04 was measured in the bioreactor after inoculation. All fermentations were supplemented with 120 μg·mL^−1^ ampicillin, 34 μg·mL^−1^ chloramphenicol, and the amylase for glucose release. After 15 h cultivation, booster tablets and amylase were added according to the manufacturer’s protocol. The pre-induction of the pMJS9 encoded gene products was carried out in the presence of 0.5% *w/v* arabinose added to the bioreactor 30 min after the booster tablets. Isopropyl-β-D-thiogalactopyranosid (IPTG) was added after another 30 min to a final concentration of 1 mmol·L^−1^ to induce expression of the glycosyltransferase HisDapGalNAcT2 [6]. Over the following 23.5 h 12 samples were taken from the bioreactor for offline analysis starting with the first sample after addition of arabinose. The samples were stored on ice during the cultivation.

### 2.5. Offline Analytics

OD_600_ was measured using the photospectrometer Ultrospec 3100 pro (Amersham Bioscience Europe, Freiburg, Germany). Acetate and glucose concentrations were determined enzymatically with the Konelab Arena XT (Thermo Scientific, Waltham, MA, USA) using an acetate kit (R-Biopharm AG, Darmstadt, Germany) and a glucose kit (Thermo Fisher Scientific, Waltham, MA, USA). Bacterial dry matter was determined by centrifugation of 5 ml cell suspension. The pellet was re-suspended in PBS for the transfer to a pre-weighed test tube. This was followed by an additional centrifugation step. The supernatant was discarded and the bacteria pellets in the test tubes were dried at 105 °C for 24 h and re-weighed.

### 2.6. Purification of Soluble Human Glycosyltransferase

A cell pellet derived from a 5 mL culture fraction was re-suspended in 630 µL extraction buffer (50 mmol·L^−1^ Tris, 300 mmol·L^−1^ NaCl, pH 8) containing 70 µL lysozyme, 1.5 µL DNAse I and 25 µL protease inhibitor (complete protease inhibitor cocktail tablet, F. Hoffmann La-Roche AG, Switzerland). The bacterial suspension was cooled on ice for 30 min and sonicated for 3 min on ice. The cell lysate was centrifuged at 4 °C, 16100× *g* for 10 min, and the supernatant was passed through a 0.45 µm filter (Merck Millipore, Darmstadt, Germany). The polyhistidine-tagged protein HisDapGalNAcT2 was purified using Ni-NTA spin columns (Qiagen, Hilden, Germany) with washing buffer (50 mmol·L^−1^ Tris, 300 mmol·L^−1^ NaCl, 20 mmol·L^−1^ imidazole) and elution buffer (50 mmol·L^−1^ Tris, 300 mmol·L^−1^ NaCl, 500 mmol·L^−1^ imidazole) adjusted to pH 8 prior to use [6]. The protein concentration in the eluent fraction was determined by employing a BCA-assay.

### 2.7. Human Glycosyltransferase Activity Assay

The activity of HisDapGalNAcT2 was determined using a glycosyltransferase activity kit (EA001, R & D Systems Europe Ltd., Abingdon, UK) as described previously [6]. The activity of each sample was determined using 0.5 µg soluble protein.

### 2.8. Online Data Collection

The multi-wavelength excitation/emission matrices (EEM) were recorded with a BioView^®^ system (Delta, Hørsholm, Denmark) equipped with a fiber optic assembly especially developed to fit into a 19 mm port. The benchtop bioreactor was equipped with a 20 cm stainless steel casing and the fluorescence sensor was inserted after autoclaving. A full EEM consists of 120 wavelength pairs with an excitation range from 270 to 550 nm and emissions recorded from 290 to 590 nm with a 20 nm interval. The scans were vectorized into 2-way arrays and used as x-data for the chemometric modeling. The gain of the fluorescence spectrometer was set to 1100 and the EEMs were recorded with a measurement interval of 5 min during fermentation. Only EEMs taken after booster addition were used for the chemometric modeling.

### 2.9. Chemometric Modeling

MATLAB version 8.4.0 (MathWorks, Natick, MA, USA) in combination with the PLS-toolbox version 7.9.5 (Eigenvector Research Inc., Manson, WA, USA) was used for chemometric modeling [28]. A detailed description is provided elsewhere in the literature [17,18,29]. The EEM data were preprocessed by background subtraction to the first scan after booster addition. Offline measured values and corresponding scans were used for the calibration of PLS regression models for the prediction of OD_600_, acetate, and glucose concentration. For the PLS model, regarding the ratio of soluble protein to dry cell weight, only samples 21 h after inoculation were used as input data for the calibration model. For the PLS model concerning the specific activity of HisDapGalNAcT2, the offline data was complemented using a double Boltzmann fitting operated in Origin 9.1G (OriginLab Inc., Northampton, MA, USA). Based on the fit, the resulting *y*-data in 30 min intervals and corresponding EEM were applied for the correlation starting with all scans recorded between 24.5 h after inoculation and harvest. The SIMPLS algorithm was used for PLSR model calibration in combination with venetian blinds as method for cross-validation applying six splits and one sample per split for all models. Three of the available datasets (run I–III) were used for calibration and cross-validation. Calculated RMSE and RMSECV were used to assess the performance of the model. All EEMs recorded after booster addition were fed to the respective PLS model for prediction. In order to evaluate the robustness of the calibration model, *x*-data recorded during an additional fermentation (run IV) was only predicted using the selected model. The predicted response variables of all fermentations were compared to the offline-determined values.

## 3. Results and Discussion

The expression of the human-derived, soluble, and functional glycosyltransferase HisDapGalNAcT2 represents the key factor in establishing this particular *E. coli* cell factory. The final purpose of this cell factory will be to enable the transfer of a GalNAc-residue to a protein substrate in vivo. The expression strategy for HisDapGalNAcT2 did follow a temporal sequence. Two chaperones (sulfhydryl oxidase Erv1p and protein disulfide isomerase PDI [6,27]) were induced via arabinose and mediated folding and disulfide bond formation of the HisDapGalNAcT2. The glycosyltransferase was induced by IPTG 30 min after the two redox folding helpers.

### 3.1. EnPresso^®^ B Batch Cultivations and Chemometric Modeling of Process Parameters

Four batch fermentations were carried out using EnPresso^®^ B medium and the *E. coli* SHuffle^®^ T7 strain expressing the recombinant glycosyltransferase HisDapGalNAcT2. EnPresso^®^ B medium consists of three main components: (1) medium tablets, (2) booster tablet and (3) amylase for controlled glucose release. The components of the tablets are not stated by the manufacturer but it is known that a polysaccharide is an ingredient of the booster and the medium tablets. Following the protocol provided by the manufacturer, the booster was added to the medium 15 h after inoculation and 1 h before the chaperones were induced. A sufficient nutrition of the cell factory was achieved by addition of amylase at the inoculation and again together with the booster [7]. The measured glucose concentrations ranged between 0.3–2.3 g·L^−1^ during the cultivations. Low cell growth with doubling times of 172 ± 4.4 min was observed during the first 15 h after inoculation prior to booster addition. The booster addition accelerated the cell growth and doubling times of 110 ± 17.8 min were observed. The growth rates declined already 2 h after booster addition and doubling times between 30–60 h within the following 20 h were observed. Nevertheless, all EnPresso^®^ B cultivations reached higher OD_600_ values at the end of the process in comparison to the cultivation with LB-medium (Figure S1).

Offline values and corresponding EEMs of three fermentations (runs I–III) were used for calibration and cross-validation of the respective soft sensors. Resulting PLSR models were selected and evaluated based on preferably low rout mean squared errors for calibration and prediction (RMSEC and RMSEP) in combination with R^2^ > 0.9 for calibration (R^2^_cal_) and validation (R^2^_CV_) if possible (Table 1). The number of latent variables (LVs) required for each model was minimized and it was aimed for a maximum of captured *x*- and *y*-variance simultaneously. The PLSR models were, furthermore, applied to predict the respective *y*-values during an additional batch cultivation using EnPresso^®^ B (run IV). This was done to investigate the robustness and predictive power of these models.

### 3.2. Overall Batch Behavior Evaluated by Principal Component Analysis

**Figure 1:**
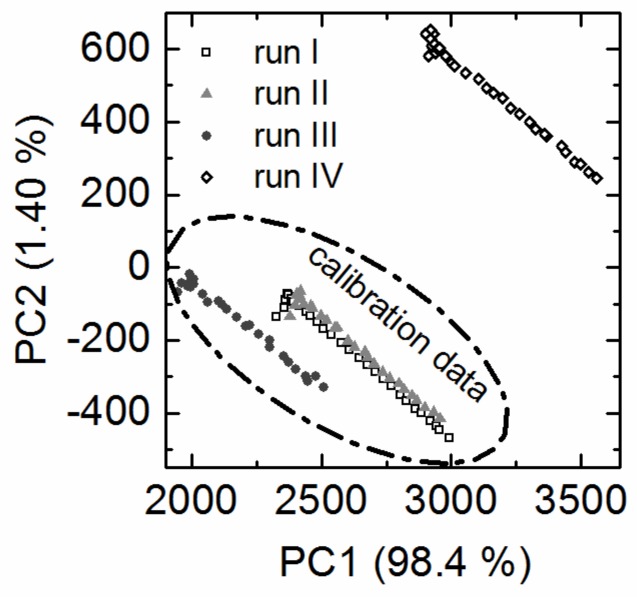
PCA scores plot. The EEM raw data was used as input for the PCA. The PCA scores plot shows the differences between the four batch cultivations with EnPresso^®^ B medium. For clarity only every tenth point is shown. The circle indicates the cultivations used for PLS model calibration and run IV was used as test dataset to predict the target parameters. The cultivation run IV was carried out with six months time lag to the other cultivations.

Principal component analysis (PCA) was used to investigate the structure of the fluorometric datasets and enabled the identification of differences between the batch cultivations with EnPresso^®^ B medium prior to PLS modeling. The relation of the individual EEMs to each other can be displayed in the PCA scores plot. EEMs with similar scores are considered similar. The score values calculated for the four fermentations formed similar trajectories on the PCA score plot (Figure 1) which were compared and put into relation. The fluorometric dataset was not preprocessed, but the scores plot showed that 99.8% of the variance in the dataset was already captured by two principal components (PC).

The similar score values of batch cultivation I and II indicated that they had the same background fluorescence. The trajectory of batch cultivation III differed mainly on PC1 from cultivation I and II. Furthermore the trajectory of cultivation IV differed on PC1 and PC2 from all other cultivations. It was suspected that lot to lot variability of the EnPresso^®^ B medium or the amylase performance might have caused this variability in the datasets. This will be discussed in the following chapters.

### 3.3. Prediction of Acetate Concentrations and Optical Density

**Figure 2:**
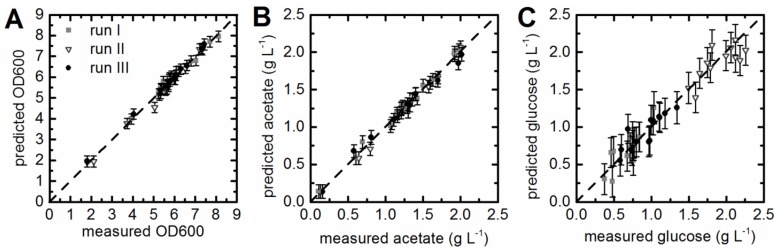
Prediction versus reference plots. PLS regression models were built for OD_600_ (**A**), acetate (**B**), and glucose concentrations (**C**). Online and offline data of three cultivations was used for calibration and cross-validation of the PLS models. Vectorized EEMs taken at the sample points (*x*-data) and the offline measured values (*y*-data) were correlated. Predicted versus reference plots of the PLS models did show good correlation.

**Figure 3:**
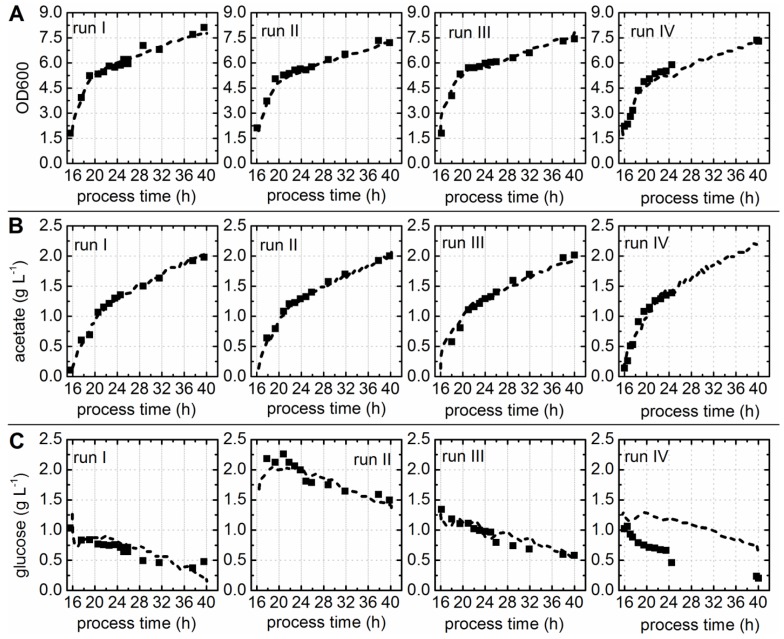
Correlation of predicted and offline measured process parameters. Predicted values (dashed) were compared to offline values (squares). Runs I–III were used for calibration and internal cross-validation of the soft sensors predicting OD_600_ (**A**), acetate (**B**), and glucose concentrations (**C**). Run IV was not included in model generation; this cultivation was used to test the selected model.

The calculated PLS models for the prediction of acetate concentrations and OD_600_ values showed in both cases a calculated R^2^_cal_ > 0.98 (Table 1). The quality of the fit was also evaluated with the help of the predicted versus measured plots (Figure 2A,B). All OD_600_ values and acetate concentrations were located closely to the target line. Captured variances of 99.9% for the x-data and 99.85% for the y-data with only four LVs were achieved for the PLS model which was built to predict acetate concentrations. In addition, 81.87% of the variance in the x-data and 89.81% of the variance in the y-data was captured for the OD_600_ model also with four LVs (Table 1). The correlation of predicted values and offline measured acetate concentrations and OD_600_ values was good for all batch cultivations used for model generation (Figure 3A,B). Occasionally samples taken during batch cultivation run IV were analyzed offline. The measured values did fit the prediction for acetate concentrations and OD_600_ (Figure 3A,B). This led to the assumption that the proposed soft sensors were reliable and allowed the online prediction of acetate concentrations and OD_600_.

### 3.4. Prediction of Substrate Concentrations

Establishing a soft sensor for the online prediction of glucose concentrations was challenging. Since the glucose supply of the culture was accomplished enzymatically through amylase (glucoamylase) [30]. The same batch of predefined EnPresso^®^ B medium was used for all cultivations. The glucose concentrations determined for samples taken from the bioreactor during batch cultivation run I and run III ranged between 0.5 g·L^−1^ and 1.5 g·L^−1^. However, the glucose concentrations measured during batch cultivation run II were higher, ranging from 1.5 g·L^−1^ up to 2.3 g·L^−1^. Despite the differences between the three cultivations concerning the measured glucose concentrations, a PLS model was calibrated and cross-validated. The resulting model with three LVs and an R^2^_cal_ of 0.94, R^2^_CV_ of 0.88, and a RMSEP of 0.19 g·L^−1^ was accepted (Table 1), since the predicted versus measured plot showed good correlation (Figure 2C). Glucose concentrations predicted for cultivations included in the model generation (run I–III) were in good accordance with the offline measured values (Figure 3C). The predicted glucose concentrations of batch fermentation run IV were roughly 1 g·L^−1^ higher than the offline measured values (Figure 3C). Nevertheless, predicted and measured values showed the same trend over cultivation time. The discrepancies between the offline measured values and predicted glucose concentration might be explained as follows: Glucose is a non-fluorescent compound, but glucose uptake and consumption by the cells has an impact on the pattern of fluorescent components in the culture. Thus, chemometric models for glucose prediction are generally based on these patterns, so-called secondary effects. The EnPresso^®^ B medium contained a polysaccharide and the glucose supply was regulated through amylase. The information about how much glucose was released over time was not available, because the glucose was continuously metabolized by the cell factory.

The observed circumstances let to the assumption that either the enzyme used in run IV or the cell metabolism behaved differently, which was already suspected based on the PCA results. First, a different enzyme lot was used for this particular cultivation. Second, the medium was stored as separately-wrapped tablets and, although the same medium lot was used, differences in the appearance of the tablets were observed due to storage. The largest difference concerning the color and the solubility in water was noticed between the medium tablets used for the first tree cultivations (runs I–III) and for cultivation IV. This validation run IV was conducted with a time lag of six months to the other cultivations.

### 3.5. Chemometric Modeling of the Cell Factory’s Efficiency

The proposed *E. coli* cell factory is a complex expression system with the objective to perform posttranslational changes to a protein substrate in vivo. Real-time monitoring of the glycosyltransferase specific activity during cultivation might be an advantage since the enzyme was not the final product of this process, but it was an indicator for the cell factory’s efficiency. Since the ability to glycosylate a protein substrate is always directly related to the concentration of functional active HisDapGalNAcT2 in the cytoplasm.

### 3.6. Prediction of Soluble Protein to Biomass Ratio

**Figure 4:**
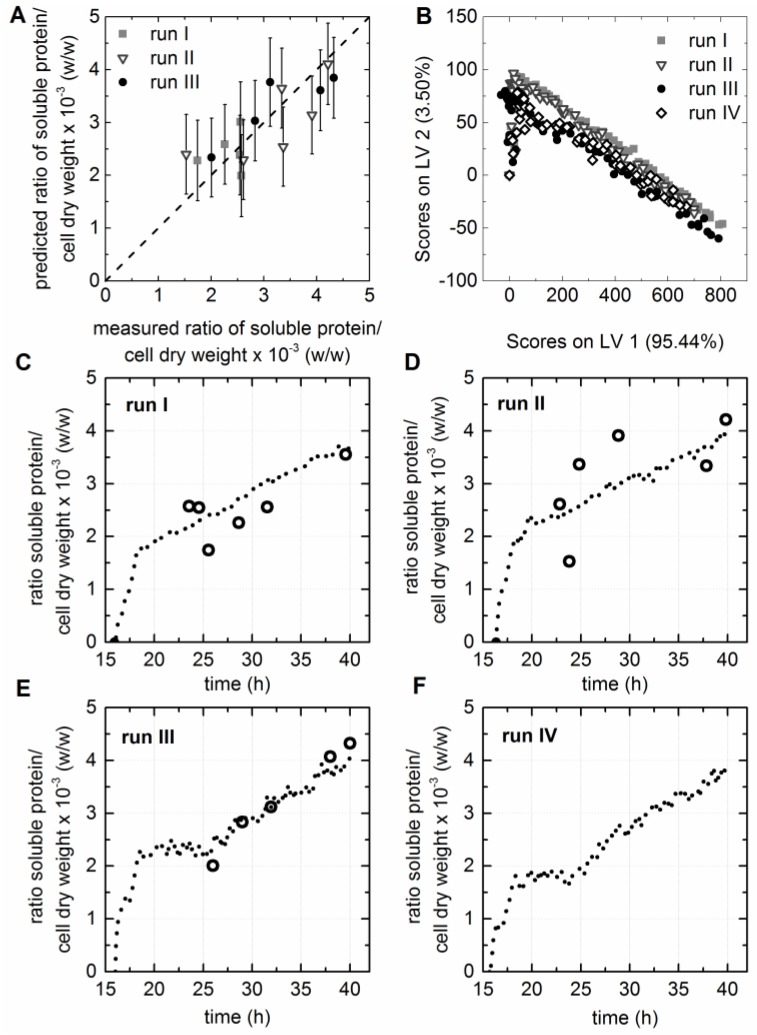
PLS model for the prediction of soluble protein to cell dry weight ratios. (**A**) The predicted versus reference plot of the PLS model with three LVs. (**B**) The scores values of all EEMs calculated for LV1 and LV2 are shown in the scores plot. (**C–F**) Offline (circles) and predicted values (dashed line) based on the selected PLS model are shown for cultivations I–IV.

A PLS regression model for the prediction of soluble protein accumulation in the cytoplasm of the cell factory was developed. Therefore, the ratio of captured soluble protein to dry cell matter was calculated and used as the *y*-value for the model calibration. It has to be considered that the offline measured soluble protein concentrations might have been biased due to the laborious purification method, since the chance to co-purify small amounts of host cell protein during the capture step with Ni-NTA spin columns was given. For this reason, only *y*-data determined for samples taken after 21 h cultivation time were used as input for the model. For these samples it was assumed that the amount of possibly co-purified host cell protein was negligible in comparison to the concentrations of the recombinant protein. The resulting calibration model with three LVs was able to capture 79.2% of the *x*-variance and 86.2% of the *y*-variance. The measured versus predicted plot of the PLS regression model showed a close correlation (Figure 4A) with correlation coefficients of R^2^_cal_ = 0.862 and R^2^_CV_ = 0.744 (Table 1). The scores plot showed that the EEMs of all four fermentations behaved similarly after preprocessing of the raw data by background subtraction (Figure 4B). This supported the supposition that the variations in the fluorescence data sets were related to the background fluorescence caused by the medium, like the PCA results, was already indicated. The PLS model was used to predict the ratio of soluble protein to cell dry weight based on the EEMs recorded during the cultivations. Figure 4C–E shows that offline and predicted values were in good accordance. The soluble protein to cell dry weight ratio was also predicted for fermentation IV (Figure 4F). However, a corresponding offline dataset was not available for this run. Nevertheless, the predicted values showed the same progression over process time as observed for all other cultivations. This indicated that the selected PLS model interpreted the fluorometric dataset of this fermentation in the same way as all other fermentations. The ratio of soluble protein to cell dry matter of all cultivations did steadily increase to approximately 4 × 10^−3^ µg (*w/w*). However, a distinct increase of the ratio was observed for the first five hours after induction. This observation can be assigned to the second glucoamylase addition prior to induction, and consequently enhanced glucose release [7]. One hour after induction, the replication of the plasmids and the synthesis of the recombinant proteins most likely effected the cell growth. Diaz and Hernández [31] showed that cell metabolism and doubling times can be influenced by various parameters, such as plasmid size (pMJS9: 8.1 kBp and pET23d(+)::*HisDapGalNAcT2*: 5.4 kBp), copy number, over expression of homologous or heterologous genes, and their size. This particular *E. coli* cell factory was genetically engineered to express three heterologous genes: (1) sulfhydryloxidase (21.6 kDa); (2) protein disulfide isomerase (58.2 kDa); and (3) glycosyltransferase (61.7 kDa). This metabolic shift might have been crucial for the formation of active human recombinant HisDapGalNAcT2. In accordance with this, Luchner et al. showed, for human superoxide dismutase expressed in *E. coli*, that the ratio of active soluble protein to its aggregated inactive form was strongly dependant on the growth rate [9]. It was concluded that the uptake and processing of the HisDapGalNAcT2 by the chaperones, might only work when the cell growth and the glycosyltransferase expression was slowed down.

### 3.7. Prediction of the Specific Activity of the HisDapGalNAcT2

**Figure 5:**
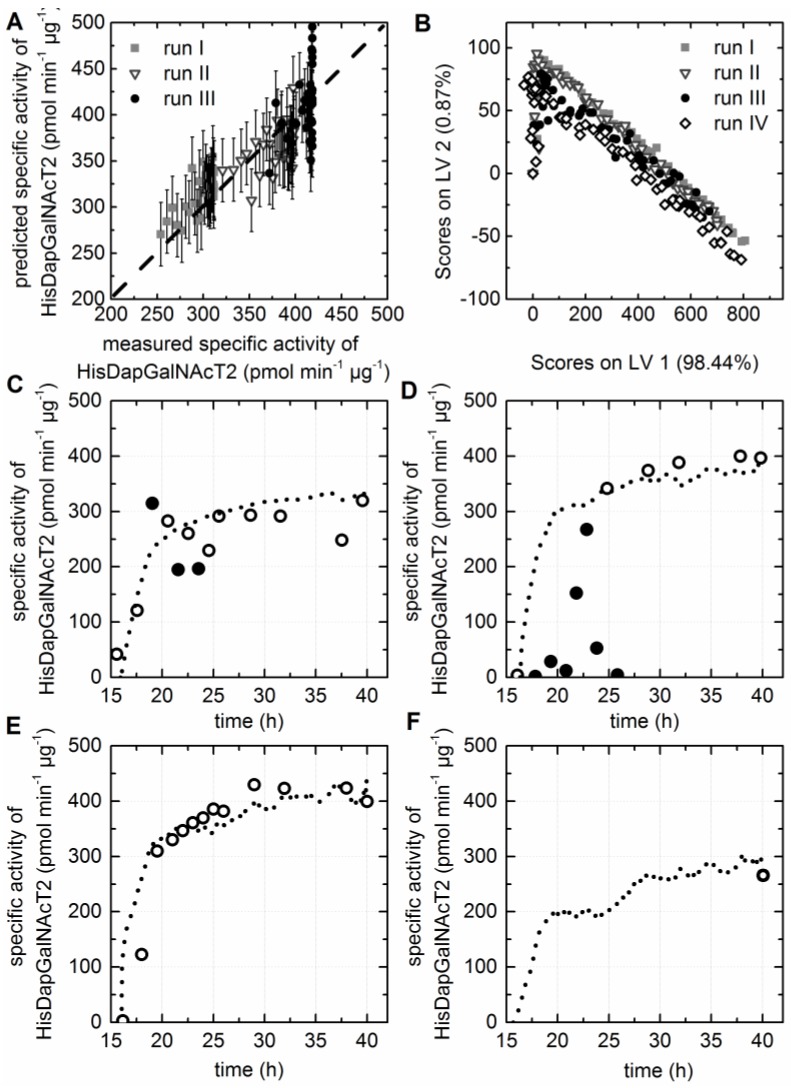
PLS model for the prediction of the specific activity of HisDapGalNAcT2. (**A**) The predicted versus reference plot of the PLS Model with three LVs. (**B**) The score values of all EEMs calculated for LV1 and LV2 are shown in the scores plot. (**C–F**) Offline (circles) and predicted (dashed line) values based on the PLS model are shown for cultivation I–IV. Only samples taken from the bioreactor with a determines OD_600_ > 5.0 were used as input data for the model, since a high number of possible outliers (closed circles) were identified for samples with lower OD_600_ values.

The specific activity of the purified HisDapGalNAcT2 was determined following the activity assay described by Lauber et al. [6]. Three LVs were selected for the resulting PLS model predicting the specific activity of the HisDapGalNAcT2. The considerably low R^2^_cal_ of 0.654 and R^2^_CV_ of 0.591 did not meet the requirements described earlier, where correlation coefficients of >0.9 were aimed for (Table 1). However, a low RMSE for calibration and prediction, together with a low number of LVs, was accomplished for the selected model. The deviation of the data from the target line in the measured versus predicted plot (Figure 5A) was smaller for runs I and II than for cultivation run III. Nevertheless, it was possible to accept the model since 99.4% of the *x*-data variance and 99.3% of the *y*-data variance was captured. The scores plot showed that the EEMs of all four fermentations behaved similar after preprocessing of the raw data by background subtraction (Figure 5B). The three cultivations which were included in the model calibration covered final specific activities from 276 to 426 pmol·min^−1^·µg^−1^. Knowing that the cell factory showed a certain biological diversity supports the need for in situ monitoring, since the offline analytics are too time-intensive, and even small variations of the process due to the medium storage or the use of a different enzyme lots had an impact on the cell factory. The specific activity of the enzyme increased strongly within the two hours after booster and glucoamylase addition (Figure 5C–F), as it was also observed for the soluble HisDapGalNAcT2 to dry matter ratio. The results showed that the extraction of soluble protein from the cell lysate followed by the activity assay was prone to errors in the case of low cell concentrations and, therefore, also low enzyme concentrations. Outliers were predominantly suspected for samples with OD_600_ values < 5. Consequentially only specific activities determined for samples with OD_600_ values > 5 were used for model calibration. It was assumed, that at this point the chaperones were expressed in a sufficient quantity to obtain the active conformation of the glycosyltransferase. This resulted in a limitation of the selected PLS model, which was already suspected due to the low correlation coefficients. The accuracy of the calibration models is always dependent on the accuracy of assay or the method used for the analysis of the respective response variable. Therefore, the prediction of difficult-to-access process parameters remains a challenge. However, the data supported the earlier described assumption of a slow growth rate and preventing the cells from a metabolism overflow supported the constant accumulation of soluble glycosyltransferase in the cytoplasm [7]. Fluorescence EEMs recorded during cultivation run IV were used to test the soft sensor regarding the online predictability of the cell factory performance. A specific activity of 280 pmol·min^−1^·µg^−1^ was determined for the HisDapGalNAcT2 at the end of cultivation run IV. Furthermore, a specific activity of 265.5 ± 3.2 pmol·min^−1^·µg^−1^ was predicted based on the PLS model and the EEM recorded during the last 30 min of the cultivation (Figure 5F). This was a promising result, since a RSMEP of 33.5 pmol·min^−1^·µg^−1^ and a RMSEC of only 30.7 pmol·min^−1^·µg^−1^ was calculated for this soft sensor (Table 1).

## 4. Conclusions

The specific activity measurements indicated that the complex and interlinked expression of glycosyltransferase and chaperones was extremely sensitive to process variations. The results suggested that the slower growth of the recombinant *E. coli* SHuffle^®^ T7 strain in EnPresso^®^ B medium slowed down the protein expression and presumably enabled the chaperone-mediated folding and disulfide-bound formation [13]. It was feasible to set up a reliable in situ monitoring for OD_600_ and acetate concentrations. To provide a PLS model for the prediction of glucose concentrations was challenging. The glucose release and therefore the glucose concentrations in the medium were not only dependent on the glucose consumption by the cells but also on the amylase activity. Furthermore, the development of a soft sensor for in situ prediction of the soluble protein content in the cells and the specific activity of HisDapGalNAcT2 was complex. One drawback in this context was that the specific activity of the enzyme had to be measured after purification from an *E. coli* cell lysate prior to PLS modeling. The results indicate that the use of more datasets might be beneficial for the calibration of such PLS models. Moreover, an improved assay for the determination of the specific activity of HisDapGalNAcT2 might facilitate the model calibration. Nevertheless, the study pointed out that time-consuming and costly offline analysis might be rendered unnecessary for complex expression systems in the near future.

## Figures and Tables

**Table 1:** Quality attributes of the PLS models for the prediction of glucose and acetate concentrations, OD_600_, the ratio of soluble protein to cell dry matter, and the specific activity of HisDapGalNAcT2.

Target	LV	R^2^_cal_	R^2^_CV_	RMSEC	RMSEP
Glucose concentration (g·L^−1^)	3	0.93	0.88	0.14	0.19
Acetate concentration (g·L^−1^)	4	0.99	0.97	0.05	0.08
OD_600_	4	0.99	0.97	0.02	0.24
Specific activity GalNAcT2 (pmol·min^−1^·µg^−1^)	3	0.65	0.59	30.7	33.5
Ratio of soluble GalNAcT2 / Dry matter (µg *w/w*)	3	0.86	0.74	4 × 10^−4^	5.5 × 10^−4^

## Supplementary Materials

The following are available online at http://www.mdpi.com/2306-5354/3/4/32/s1, Figure S1: *E. coli* Shuffle^®^ T7 cell factory LB-medium fed-batch process. OD_600_, as well as glucose and acetate, concentrations were measured offline. The glucose target concentration for the feed was 1 g·L^−1^. The cell factory was induced following the same protocol as described for the EnPresso^®^ B medium. Only negligible amounts of glycosyltransferase were formed and the fermentation was stopped after increased inclusion body accumulation was observed and the *E. coli* cell morphology changed.
