# Supplementary material for: Fluorometric In Situ Monitoring of an Escherichia coli Cell Factory with Cytosolic Expression of Human Glycosyltransferase GalNAcT2: Prospects and Limitations
Source: Bioengineering (Basel). 2016 Nov 21;3(4):32. doi: 10.3390/bioengineering3040032 (PMC5597275; doi:10.3390/bioengineering3040032)
Supplement: Supplementary file 1 [file bioengineering-03-00032-s001.pdf]

bioengineering

Supplementary Materials: Fluorometric In Situ Monitoring of an *Escherichia coli* Cell Factory with Cytosolic Expression of Human Glycosyltransferase GalNAcT2: Prospects and Limitations

Karen Schwab *,†, Jennifer Lauber † and Friedemann Hesse

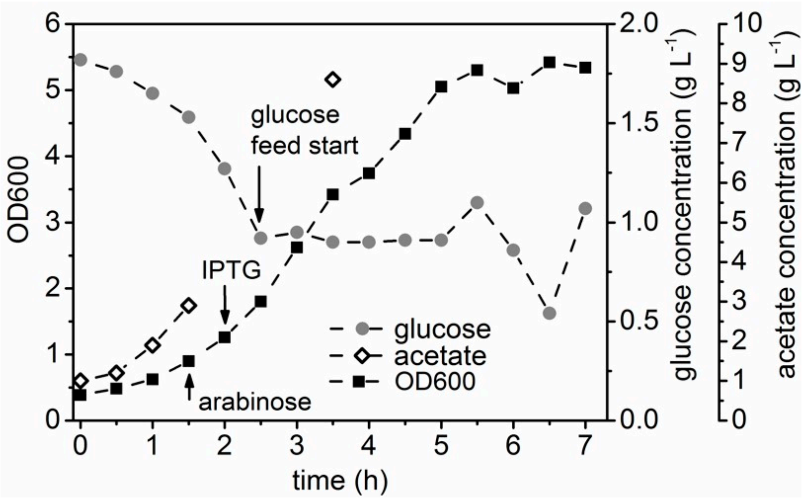

Figure S1. *E. coli* Shuffle® T7 cell factory LB-medium fed-batch process. OD_{600}, as well as glucose and acetate, concentrations were measured offline. The glucose target concentration for the feed was 1 g L^{-1}. The cell factory was induced following the same protocol as described for the EnPresso® B medium. Only negligible amounts of glycosyltransferase were formed and the fermentation was stopped after increased inclusion body accumulation was observed and the *E. coli* cell morphology changed.

© 2016 by the authors. Submitted for possible open access publication under the terms and conditions of the Creative Commons Attribution (CC-BY) license (http://creativecommons.org/licenses/by/4.0/).
